# *Agaricus bisporus* Extract Exerts an Anti-Obesity Effect in High-Fat Diet-Induced Obese C57BL/6N Mice by Inhibiting Pancreatic Lipase-Mediated Fat Absorption

**DOI:** 10.3390/nu15194225

**Published:** 2023-09-30

**Authors:** Hyungkeun Kim, Young-Eun Jeon, So-Mi Kim, Jae-In Jung, Donghyeon Ko, Eun-Ji Kim

**Affiliations:** 1 Department of Food Business, SAMOH Pharm Co., Ltd., Seoul 06244, Republic of Korea; hkkim@samohpharm.co.kr (H.K.); dhko@samohpharm.co.kr (D.K.); 2 Industry Coupled Cooperation Center for Bio Healthcare Materials, Hallym University, Chuncheon 24252, Republic of Korea; yeye@hallym.ac.kr (Y.-E.J.); somisss@hallym.ac.kr (S.-M.K.); jungahoo@hallym.ac.kr (J.-I.J.)

**Keywords:** *Agaricus bisporus*, chitosan, polysaccharide, lipid absorption, pancreatic lipase, obesity

## Abstract

*Agaricus bisporus* is well known as a source of polysaccharides that could improve human health. The objective of this study was to explore the anti-obesity effect of *A. bisporus* extract (ABE), abundant in polysaccharides, and its underlying mechanism. Pancreatic lipase inhibitory activity in vitro was determined after treatment with ABE and chitosan. Treatment with ABE and chitosan significantly decreased pancreatic lipase activity. Five-week-old male SD rats were randomly divided into three groups for acute feeding with vehicle, ABE at 80 mg/kg body weight (BW)/day, and ABE at 160 mg/kg BW/day. ABE dose-dependently increased plasma lipid clearance in an oral lipid tolerance test. Five-week-old male C57BL/6N mice were fed a control diet (CD), a high-fat diet (HFD), an HFD with ABE at 80 mg/kg BW/day, ABE at 160 mg/kg BW/day, or chitosan at 160 mg/kg BW/day for eight weeks. HFD-fed mice showed significant increases in body weight, fat mass, white adipose tissue, average lipid droplet size, and serum levels of glucose, triglyceride, ALT, and AST compared to those in the CD group. However, ABE or chitosan administration ameliorated these increases. ABE or chitosan significantly reduced dietary efficiency and increased fecal excretion levels of lipids, triglycerides, and total cholesterol. These in vitro and in vivo findings suggest that ABE might act as an anti-obesity agent by inhibiting pancreatic lipase-mediated lipid absorption, at least in part.

## 1. Introduction

Obesity is a complex medical condition characterized by excessive accumulation of body fat due to consumption of high-calorie foods coupled with a sedentary lifestyle. According to the World Health Organization (WHO), being overweight or obese is defined as having a body mass index (BMI) equal to or greater than 30 kg/m^2^. The global obesity prevalence has increased. It is expected to continue increasing [1,2,3]. According to statistics, the global level of those who are overweight and obese reached 38% in 2020. It is anticipated to reach around 50% by 2035 [4]. Being considered a chronic disease, obesity is also known as a risk factor for metabolic disease. It can lead to the development of chronic diseases such as diabetes, cardiovascular diseases, and nonalcoholic fatty liver disease (NAFLD) [5,6]. Although a number of nature-derived ingredients have entered the market, there is still a need for efficient ingredients to reduce obesity with less adverse effects [2,3,7]. 

The development of preventive and therapeutic agents against obesity is based on five different mechanisms of action, including stimulating thermogenesis, lowering lipogenesis, enhancing lipolysis, suppressing appetite, and decreasing lipid absorption [8]. Among these mechanisms, decreasing lipid absorption is recognized as having the lowest adverse effects with limited application specifically in the duodenum rather than in the blood or brain [8,9]. Orlistat (tetrahydrolipstatin) isolated from *Streptomyces toxytricini* is known as an efficient anti-obesity reagent with approval by United States Food and Drug Administration (FDA) [10]. However, its adverse effects (such as steatorrhea, hepatotoxicity, kidney injury, osteoporosis, and oncogenesis) and interactions with drugs (such as warfarin, amiodarone, and thyroxine) and fat-soluble vitamins should not be overlooked [10,11]. Natural extracts concentrated in bioactive compounds can serve as good candidates for safe anti-obesity reagents by targeting lipid absorption [12,13,14]. 

*Agaricus bisporus*, commonly known as white button mushroom, is a type of edible mushroom that is widely consumed around the world. This mushroom is a good source of nutrients including protein, fiber, vitamins B and D, minerals, and bioactive compounds such as polysaccharides and polyphenols [15]. *A. bisporus* has been studied for its potential health benefits including anticarcinogenic, antimicrobial, antioxidant, anti-inflammatory, and immunomodulatory activities [16,17]. Various kinds of bioactive compounds such as glycoprotein, β-glucan, chitin, and chitin derivatives are present in the cell walls of *A. bisporus*. Different *A. bisporus* extraction methods can result in differential biological effects [17,18]. Currently, clinical trial results have revealed that *A. bisporus* extract with a high density of polysaccharides can exert an anti-obesity effect [19]. However, its exact mechanism of action has not been defined yet. Several studies have reported that *A. bisporus* can exert anti-obesity effects through beta-oxidation and autophagy, although such effects depend on the composition of the *A. bisporus* extract [20,21]. 

In the present study, we attempted to investigate the anti-obesity effect of *A. bisporus* extract (ABE) containing a high density of polysaccharides with a unique extraction and concentration method [18]. Possible mechanisms underlying this effect were also explored. For such purpose, we determined the effect of ABE on pancreatic lipase activity in vitro and oral lipid tolerance in Sprague–Dawley (SD) rats. Furthermore, we investigated the potential in vivo effect of ABE on body fat accumulation and obesity-related biomarkers in high-fat diet-induced obese C57BL/6N mice. Our results suggest that ABE might act as an anti-obesity agent by inhibiting pancreatic lipase-mediated fat absorption, at least in part.

## 2. Materials and Methods

### 2.1. Preparation of ABE

ABE, which contains a high density of polysaccharides (H2Oslim^®^), was generously supplied by Tradichem SL (Madrid, Spain). In a brief overview of the extraction process, ABE was extracted using a 1:10 ratio of *A. bisporus* to distilled water, with the addition of a deacetylation agent, NaOH. The extraction was subsequently neutralized by the addition of HCl. The insoluble components of the extraction were removed through filtration, resulting in the isolation of a highly concentrated polysaccharide ABE. This concentrated ABE was then made into a powder form using spray drying. The final yield ranged from 3 to 7%.

### 2.2. Determination of Chitosan Contents in ABE

Chitosan content of ABE was analyzed by measuring the content of total D-glucosamine following the analytical method listed in the Korean Health Functional Food Codex. Hydrolysis with hydrochloric acid (Junsei Chemical Co., Ltd., Tokyo, Japan) followed by quantification of glucosamine is a commonly used analytical method for chitosan quantification [22,23]. Briefly, ABE was hydrolyzed using hydrochloric acid, resulting in the production of glucosamine. This glucosamine further reacted with acetylacetone (Junsei Chemical Co., Ltd.) under alkaline conditions, leading to the formation of chromogen 2-methyl-3-diacetylpyrrole derivatives. When the chromogen was exposed to p-Dimethylaminobenzaldehyde (Sigma-Aldrich Co. St. Louis, MO, USA) under acidic conditions, a purplish red compound was formed. The absorbance of this compound was measured at a wavelength of 530 nm. Within a specific concentration range, the absorbance was directly proportional to the concentration of glucosamine (Sigma-Aldrich Co., St. Louis, MO, USA), allowing for its quantification using spectrophotometry (Thermo Fisher Scientific, Vantaa, Finland). Total chitosan content was calculated using Equation (1):

Chitosan content as total glucosamine (mg/g) = (Sc × Ta × 10)/(Sa × Tw × 1000)(1)

where Sc is the total glucosamine concentration in the standard (μg/mL), Ta is the absorbance of test material, Sa is the absorbance of standard, and Tw is the weight of test material (g).

### 2.3. In Vitro Pancreatic Lipase Activity Assay

Pancreatic lipase inhibitory activity was assayed using a porcine pancreatic lipase and *p*-nitrophenyl butyrate (*p*-NPB) as a substrate according to a method reported previously [24]. An enzyme buffer was prepared by adding porcine pancreatic lipase (Sigma-Aldrich Co., St. Louis) solution reconstituted with 10 mM morpholine propanesulfonic acid (MOPS) and 1 mM ethylenediaminetetraacetic acid (EDTA) (pH 6.8) to Tris buffer (100 mM Tris-HCl and 5 mM CaCl_2_, pH 7.0). Then, ABE at various concentrations was mixed with enzyme buffer and incubated at 37 °C for 15 min. After incubation, 10 mM *p*-NPB was added, and the enzyme reaction was allowed to proceed at 37 °C for 15 min. Pancreatic lipase activity was determined by measuring hydrolysis of *p*-NPB to *p*-nitrophenol at 400 nm. Inhibition of pancreatic lipase activity (%) was calculated as (1 − (Absorbance of test sample with enzyme − Absorbance of test sample without enzyme)/Absorbance of Blank) × 100. 

### 2.4. In Vivo Oral Lipid Tolerance Test (OLTT)

The animal study protocol was approved by the Institutional Animal Care and Use Committee of Hallym University (approved number: Hallym 2023-2). The animal study was conducted following the guidelines for the care and use of laboratory animals.

Five-week-old male Sprague–Dawley (SD) rats were purchased from Dooyeol Biotech Co., Ltd. (Seoul, Republic of Korea) and kept at the animal research facility of Hallym University. They were maintained at 23 ± 3 °C and 50 ± 10% relative humidity with a 12 h light/dark cycle. During the acclimation period for one week, rats had free access to a commercial, non-purified rodent diet and tap water. 

After the one-week acclimation period, rats were randomly allocated into three groups (n = 10 per group) as follows: (i) lipid emulsion control group (LC), (ii) lipid emulsion + 80 mg/kg body weight (BW) ABE group (L + A80), and (iii) lipid emulsion + 160 mg/kg BW ABE group (L + A160). After fasting for 16 h, rats in L + A80 and L + A160 groups were orally administered ABE at doses of 80 and 160 mg/kg BW, respectively. Rats in the LC group were orally administered sterile water as a vehicle. After 10 min, rats were administered a lipid emulsion consisting of 200 g/L soybean oil, 12 g/L lecithin from soybean, and 22.5 g/L glycerol using an oral gavage at a dose of 10 mL/kg BW. All rats were anesthetized with isoflurane (Vspharm, Hanam, Republic of Korea). Blood was collected from the orbital vein before (0 h) and at 1, 2, 4, and 6 h after administration of the lipid emulsion. Concentrations of triglyceride and total cholesterol in serum were measured using ASAN SET TG-S and ASAN SET Total-Cholesterol kits (ASAN PHARM Co., Ltd., Hwaseong, Republic of Korea), respectively, according to the manufacturers’ instructions. The area under the concentration–time curve (AUC) of triglyceride or total cholesterol was calculated according to the trapezoid rule to evaluate exposure that integrated concentration across time.

### 2.5. Experimental Design in High-Fat Induced Obesity Animal Models

The animal study protocol was approved by the Institutional Animal Care and Use Committee of Hallym University (approved number: Hallym 2023-2). The animal study was conducted following guidelines for the care and use of laboratory animals.

Four-week-old male C57BL/6N mice were purchased from Dooyeol Biotech Co., Ltd. (Seoul, Republic of Korea). They were kept at the animal research facility of Hallym University. They were maintained at 23 ± 3 °C and 50 ± 10% relative humidity with a 12 h light/dark cycle. During the acclimation period for one week, mice had free access to a commercial, non-purified rodent diet and tap water.

After a one-week acclimation period, mice were randomly allocated into five groups (n = 10 per group) as follows: (i) control diet group (CD), (ii) high-fat diet group (HFD), (iii) high-fat diet + 80 mg/kg BW/day ABE group (HFD + A80), (iv) high-fat diet + 160 mg/kg BW/day ABE group (HFD + A160), and (v) high-fat diet + 160 mg/kg BW/day chitosan group (HFD + C160). Mice in the CD group were fed a control diet (with 10% kcal of fat; cat. no. D12450B, Research Diets, Inc., New Brunswick, NJ, USA). Mice in other groups were fed a high-fat diet (with 60% kcal of fat; cat. no. D12492, Research Diets, Inc.). The composition of control diet and high-fat diet used in this study are shown in Table 1. Food and water were provided ad libitum during the experiment. ABE or chitosan dissolved in distilled water was administered daily using an oral gavage for eight weeks. An equal volume of distilled water was orally administered to mice In CD and HFD groups. Food intake was measured daily, and body weight was measured weekly during the entire experimental period.

At the termination of experiment, mice were anesthetized with tribromoethanol diluted with amyl alcohol. Blood was then drawn from the orbital vein and serum was subsequently separated from the blood by centrifugation at 1500× *g* rpm for 20 min at 4 °C. After blood collection, mice were euthanized by cervical dislocation and four areas (epididymal, retroperitoneal, mesenteric, and inguinal) of white adipose tissue (WAT) were quickly excised, rinsed with physiological saline, and weighed.

### 2.6. Body Composition Assessment

One day before terminating the experiment, percentages of lean mass and fat mass were estimated using dual-energy X-ray absorptiometry (DEXA, PIXImus^TM^, GE Lunar, Madison, WI, USA).

### 2.7. Serum Biochemical Analysis

Serum levels of glucose, triglycerides (TG), and total cholesterol and activities of aspartate aminotransferase (AST) and alanine aminotransferase (ALT) in the serum were measured with a blood chemistry autoanalyzer (KoneLab 20XT, Thermo Fisher Scientific).

### 2.8. Histological Analysis

Epididymal adipose and liver tissues were fixed with 4% paraformaldehyde in phosphate buffer (0.5 M, pH 7.4), embedded in paraffin, and sectioned to a thickness of 5 μm. These tissue sections were then stained with hematoxylin and eosin (H&E). To observe fat deposition, liver tissues were embedded in Tissue-Tek OCT compound, serially sectioned to a thickness of 5 μm, and stained with oil-red O. Stained tissues were examined and photographed under a light microscope (AxioImager, Carl Zeiss, Jena, Germany) at 200× magnification. Adipocyte size in epididymal adipose tissue was analyzed using an AxioVision Imaging System (Carl Zeiss). 

### 2.9. Measurement of Lipids in Livers and Feces

Total lipids from liver tissues and feces were extracted according to a method described previously [25] with a slight modification. Briefly, each sample was mechanically homogenized in phosphate buffered saline. Chloroform–methanol (2:1, *v*/*v*) was then added and the sample was homogenized. The homogenate was centrifuged at 2500× *g* for 10 min. The upper phase was discarded. The lower chloroform phase containing lipids (triglyceride and cholesterol) was collected and evaporated in a rotary evaporator under vacuum. The lipid weight was measured. The lipid was dissolved in isopropanol and contents of triglyceride and total cholesterol were measured using a commercial kit (ASAN PHARM Co., Ltd.).

### 2.10. Statistical Analysis

All data are expressed as mean standard error of the mean (SEM). The statistical difference was evaluated using a one-way analysis of variance (ANOVA) with a post-hoc test (Duncan’s multiple range test) using SAS for Windows version 9.4 (SAS Institute, Cary, NC, USA). *p* < 0.05 was considered significant.

## 3. Results

### 3.1. Characterization of ABE 

**Figure 1:**
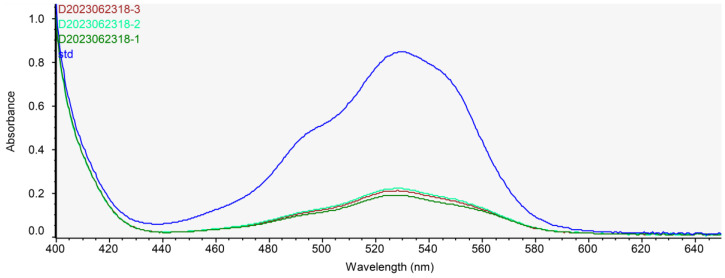
Quantitative confirmation of chitosan in ABE. Spectrophotometric profiles of D-glucosamine hydrolysate (Standard, 378.23 µg/mL) and ABE (Sample ID 1~3: 384, 450, and 424 µg/mL, respectively).

To determine contents of chitosan in ABE, ABE was analyzed in triplicate using a spectrophotometer. Results are shown in Figure 1. ABE contained 212.42 ± 0.80 mg/g of chitosan. 

### 3.2. ABE Inhibits Pancreatic Lipase Activity In Vitro

To investigate effects of ABE on fat absorption in vitro, the inhibitory effect of ABE on lipase activity was estimated using a porcine pancreatic lipase with *p*-NPB as a substrate. Results showed that ABE, with increasing concentrations of 0.01 to 1.00 mg/mL, markedly inhibited pancreatic lipase activity, ranging from 21.4% to 36.9% (Table 2). 

### 3.3. ABE Reduces Elevation of Serum Triglyceride and Cholesterol Levels after Oral Administration of Lipid Emulsion

**Figure 2:**
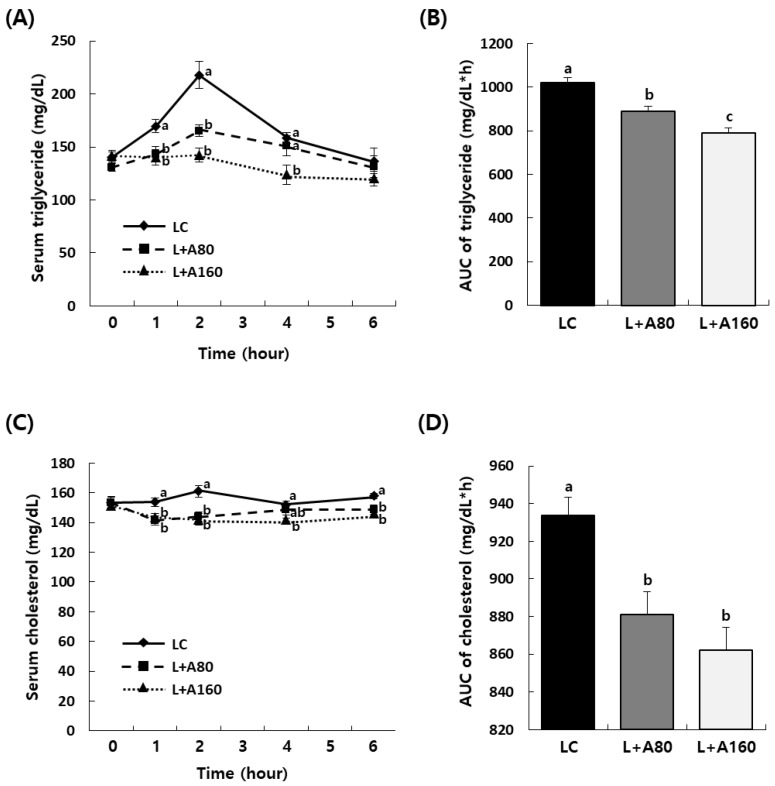
Effect of ABE administration on serum triglyceride and total cholesterol concentrations after oral administration of lipid emulsion in SD rats. SD rats were orally administered with ABE After 10 min, rats were given lipid emulsion at a dose of 10 mL/kg BW by oral gavage. Blood was collected at 0, 1, 2, 4, and 6 h after lipid emulsion administration and serum was obtained from blood. Triglyceride (**A**) and total cholesterol (**C**) concentrations in serum were measured using relevant assay kits. AUCs for serum triglyceride (**B**) and total cholesterol (**D**) were calculated. Each bar represents the mean ± SEM (n = 10). Different letters indicate significant differences between LC, L + A80, and L + A160 groups at *p* < 0.05.

OLTT was conducted to investigate whether ABE could reduce postprandial hyperlipidemia in vivo. Serum triglyceride and cholesterol levels in L + A80 and L + A160 groups during OLTT were significantly decreased at 1 h and 2 h compared to those in the LC group (*p* < 0.05) (Figure 2A,C). AUCs of triglyceride and total cholesterol were significantly reduced in L + A80 and L + A160 groups compared to those in the LC group (*p* < 0.05) (Figure 2B,D).

### 3.4. ABE Reduces Body Weight Gain and Body Fat Deposition in HFD-Induced Obese C57BL/6N Mice

ABE exhibited inhibitory effects on pancreatic lipase activity in vitro and postprandial hyperlipidemia in vivo. Based on these results, we further investigated the effect of long-term administration of ABE on obesity in an HFD-induced obese mouse model. Mushroom derived-chitosan, known as an agent that could inhibit body fat absorption, was used as a positive control. Mice fed with HFD had dramatically increased body weight gain and body fat mass with decreased lean mass compared to mice fed with a control diet (all *p* < 0.001). However, administration of ABE or chitosan significantly decreased body weight gain and body fat mass (*p* < 0.05) and increased lean mass (*p* < 0.05). In the HFD + A160 group, body weight gain and body fat mass were reduced by 36.0%, 9.3%, respectively, and lean mass was increased by 6.1% compared to those in the HFD group (all *p* < 0.05) (Table 3). 

Food efficiency ratio in the HFD group was significantly higher than that in the CD group (*p* < 0.001). ABE and chitosan administration significantly lowered food efficiency ratio (*p* < 0.05). Food efficiency ratios in HFD + A80, HFD + A160, and HFD + C160 groups were reduced by 22.3%, 29.7%, and 24.3%, respectively, compared to the HFD group (Table 3).

### 3.5. ABE Ameliorates Serum Glucose and Lipids Levels in HFD-Induced Obese C57BL/6N Mice

As shown in Table 4, all serum biochemical parameters including glucose, triglyceride, total cholesterol, ALT, and AST were significantly increased in the HFD group compared to those in the CD group. Serum glucose levels were significantly reduced in HFD + A80 and HFD + A160 groups compared to the HFD group (*p* < 0.05). Compared to the HFD group, serum levels of triglyceride were significantly reduced in both HFD + A160 and HFD + C160 groups (*p* < 0.05). However, ABE administration did not affect serum level of total cholesterol elevated by HFD feeding. ALT and AST activities elevated by HFD feeding in serum were significantly reduced in both HFD + A80 and HFD + A160 groups compared to the HFD group (*p* < 0.05). In the HFD + A160 group, ALT and AST activities were reduced by 54.2% and 27.5%, respectively, compared to the HFD group (Table 4).

### 3.6. ABE Decreases White Adipose Tissue Deposition in HFD-Induced Obese C57BL/6N Mice

**Figure 3:**
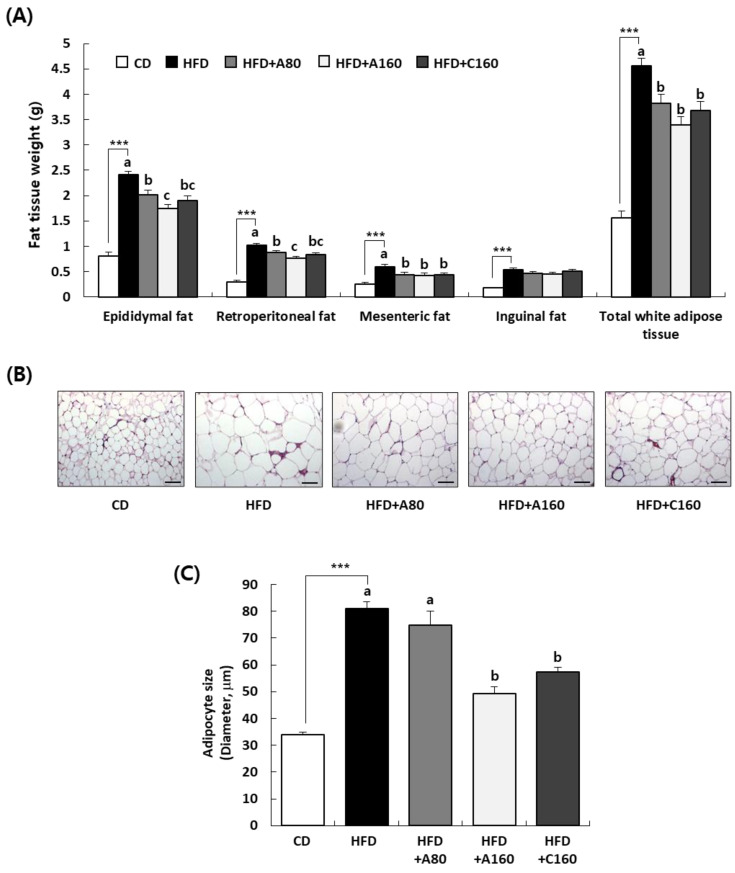
Effects of ABE administration on adipose tissue weight and morphological changes in epididymal adipose tissues of HFD-fed HFD C57BL/6N mice. Mice fed HFD were treated with ABE using an oral gavage for eight weeks. (**A**) Adipose tissue weights in epididymal, retroperitoneal, mesenteric, and inguinal fat. Total white adipose tissue (WAT) weights calculated as the sum of epididymal, retroperitoneal, mesenteric, and inguinal fat. (**B**) Extracted epididymal adipose tissues were fixed, embedded in paraffin, and cut into 5 μm thick slices. Tissue sections were stained with H&E. Representative H&E-stained images of epididymal adipose tissue (n = 5, 200× magnification) are shown. Scale bar, 50 μm. (**C**) The size of the adipocytes was quantified by measuring the longest diameter of adipocytes. Each bar represents the mean ± SEM (n = 10). *** *p* < 0.001 significantly different from the CD group. Different letters indicate significant differences between HFD, HFD + A80, HFD + A160, and HFD + C160 groups at *p* < 0.05.

To investigate whether the weight-reducing effect of ABE was due to a decrease in fat deposition in HFD-induced obese mice, the weight of adipose tissue was estimated. HFD feeding led to a significant increase in the weight of WAT (epididymal, retroperitoneal, mesenteric, and inguinal fat) (*p* < 0.001). Administration of ABE or chitosan significantly decreased weights of total WAT, epididymal fat, retroperitoneal fat, and mesenteric fat (*p* < 0.05) without affecting the weight of inguinal fat. In the HFD + A160 group, weights of total WAT, epididymal fat, retroperitoneal fat, and mesenteric fat were decreased by 25.7%, 27.8%, 25.2%, and 27.1%, respectively, compared to the HFD group (Figure 3A).

To determine whether ABE inhibited the hypertrophy of adipocytes, histological analysis of the epididymal adipose tissue was conducted using H&E staining. The HFD group exhibited larger adipocyte sizes compared to the CD group. Sizes of adipocytes were significantly decreased in HFD + A160 and HFD + C160 groups compared to the HFD group (*p* < 0.05) (Figure 3B,C).

### 3.7. ABE Inhibits Fat Accumulation in Livers of HFD-Induced Obese C57BL/6N Mice

**Figure 4:**
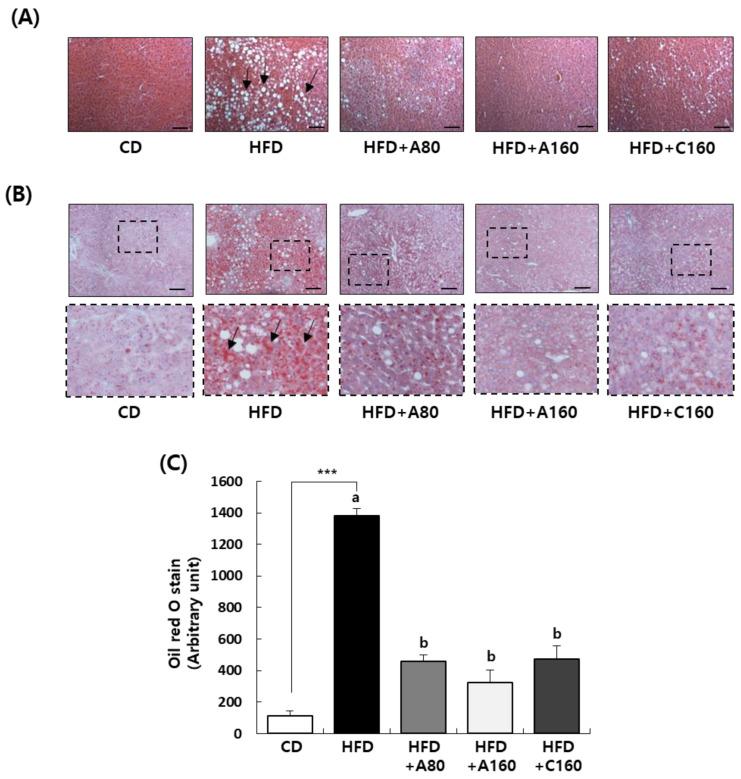
Effect of ABE administration on fat accumulation in livers of HFD-fed HFD C57BL/6N mice. Mice fed HFD were treated with ABE using an oral gavage for eight weeks. (**A**) Extracted liver tissues were fixed, embedded in paraffin, and cut into 5 μm thick slices. Tissue sections were stained with H&E. Representative H&E-stained images of liver tissue (n = 5, 200× magnification) are shown. (**B**) Liver tissues were embedded in Tissue-Tek OCT compound, serially sectioned to a thickness of 5 μm, and stained with oil-red O. Representative oil-red O-stained images of liver tissue (n = 5, 200× magnification) are shown. Scale bar: 50 μm. (**C**) Quantitative analysis of oil-red O-stained images of liver tissue. *** *p* < 0.001 significantly different from the CD group. Means without a common letter differ among the HFD, HFD + A80, HFD + A160, HFD + C160 group at *p* < 0.05.

To determine effects of ABE and chitosan on hepatic steatosis, histological observations of liver were conducted by performing H&E staining and oil-red O staining. As seen in H&E staining images of liver tissue, the HFD group revealed numerous macrovesicles and lipid droplets throughout the liver lobe compared to the CD group with normal physiological architecture of the liver, whereas administration of HFD + A80, HFD + A160, and HFD + C160 noticeably reversed these effects (Figure 4A). Moreover, oil-red O staining demonstrated considerable fat accumulation in liver sections of the HFD group, which was reduced by administration of ABE or chitosan (Figure 4B,C). For quantifying liver weight and fat accumulation in the liver tissue, we measured liver weight and levels of lipids, including triglycerides and total cholesterol, in the liver. HFD feeding considerably increased liver weight (*p* < 0.01), total content of lipids (*p* < 0.01), triglyceride (*p* < 0.05), and total cholesterol (*p* < 0.05) in the liver. Administration of ABE or chitosan significantly decreased liver weight and total lipid contents in the liver compared to the HFD group (*p* < 0.05). Contents of triglycerides and total cholesterol in the liver were decreased in HFD + A160 and HFD + C160 groups compared to the HFD group (*p* < 0.05) (Table 5). 

### 3.8. ABE Increases Fat Excretion in Feces of HFD-Induced Obese C57BL/6N Mice

ABE reduced the food efficiency ratio in HFD-induced obese mice, consistent with its inhibitory effect on pancreatic lipase activity in vitro and postprandial hyperlipidemia an OLTT model. To further investigate the effect of ABE on absorption of fats in HFD-induced obese mice, levels of fat excretion in feces, including total lipids, triglycerides, and total cholesterol, were measured. The HFD group showed significantly higher total lipid contents in feces compared the CD group (*p* < 0.001). However, there was no significant difference in the content of triglycerides or total cholesterol in feces between CD and HFD groups. Contents of total lipids and triglycerides in feces of the HFD + A160 group were significantly higher than those in the HFD group (*p* < 0.05). However, contents of total cholesterol in feces were not affected by HFD feeding and/or ABE administration (Table 6). 

## 4. Discussion

Growing evidence suggests that the use of nature-derived ingredients with specific manufacturing processes could be a promising approach for the prevention and treatment of multiple diseases [12,13,14]. *A. bisporus* contains various nutrients, including essential and semi-essential amino acids; unsaturated fatty acids; proteins; vitamins; antioxidants such as phenolic compounds, flavonoids, and tocopherols; and polysaccharides [15]. Along with its diverse range of nutrients, *A. bisporus* extract is well known for its antioxidant and immunomodulatory activities, cardiovascular health benefits, weight management properties, and positive effects on digestive health [16,17]. Several studies have investigated the anti-obesity effects of *A.bisporus* and their target molecules varied according to the respective manufacturing methods. Li et al. [20] revealed that beta-glucan-concentrated *A. bisporus* extract can exert an anti-obesity effect through PPARγ-mediated autophagy. In addition, Maria et al. [21] demonstrated that *A. bisporus* extract has an anti-obesity effect by promoting hepatic free fatty acid beta-oxidation. 

Contents and types of polysaccharides in *A. bisporus* extract vary depending on its deacetylation process. *A. bisporus* extract contains polysaccharides such as glucan, chitin, and chitosan [15,26,27]. Among these polysaccharides, chitosan is a type of dietary fiber that is derived from chitin. It is composed of randomly distributed β-(1,4) D-glucosamine and N-acetyl-D-glucosamine with a carbohydrate polymer. The deacetylation degree in chitosan, which indicates the presence of protonated -NH2 group, varies and affects its solubility [28,29]. Despite the widespread utilization of dietary chitosan for body fat reduction supported by experimental results globally, there remains a lack of consensus due to conflicting findings [30,31]. As previously reported [19,32], we obtained water-soluble polysaccharides containing ABE using our extraction and deacetylation method and mushroom-derived chitosan to confirm its biological and physiological effects on body fat reduction. The content of chitosan in the ABE was 248.96 mg/g when extracted with our method (Figure 1).

It has been previously demonstrated that the cationic polymer formed by polyelectrolyte complexation of water-soluble polysaccharides from ABE can form complexes with lipids, referred to as fat trapping ability [19,32,33]. In the present study, we examined the inhibitory effect of ABE on pancreatic lipase known to play an important role in digestion and absorption of triglycerides [34]. As the concentration of ABE increased, there was a significant inhibition of pancreatic lipase activity, with the peak effect observed at 1.00 mg/mL and maintaining as similar inhibitory effect up to 3.00 mg/mL (Table 2). It is widely recognized that orlistat covalently binds to the serine residues within the active sites of lipases, subsequently rendering them inactive. This particular mechanism of action makes orlistat’s inhibitory impact on pancreatic lipase highly potent and irreversible, thereby leading to adverse effects such as abdominal discomfort, fecal incontinence, and steatorrhea [35,36]. In contrast, ABE appears to affect the inhibition of pancreatic lipase activity by forming polymer–lipid complexes [32]. This results in a comparatively milder inhibitory effect on pancreatic lipase activity, as our study has indicated. Based on previous clinical trials [19] and pancreatic lipase activity tests, it would be assumed that the consumption of ABE is likely to induce fewer adverse effects when compared to direct inhibitory agents like orlistat. Additionally, we assessed the fat trapping ability of ABE in vivo using an oral lipid tolerance test (OLTT), a standardized method to evaluate the body’s ability to digest and absorb dietary fats [37]. Administration of ABE dose-dependently reduced both AUC and maximum plasma levels of postprandial triglyceride and cholesterol, showing statistically significant differences compared to the LC group (Figure 2). These findings suggest that ABE may suppress lipid levels by modulating lipid metabolism by reducing pancreatic lipase activity, at least in part. However, to clarify the direct effect of ABE on pancreatic lipase-mediated decreases in lipid levels in vivo, further in vivo models are still needed.

Our previous research indicated that long-term administration of ABE has preventive effects on obesity and hyperlipidemia in a randomized, double-blind, and placebo-controlled clinical trial involving overweight participants [19]. A high-fat diet (HFD)-induced obese mouse model is commonly used to study metabolic syndrome, including obesity, hyperglycemia, and hyperlipidemia [38,39]. In our study, C57BL/6 mice with HFD-induced obesity were treated with ABE for eight weeks. We observed that ABE administration suppressed the increase in body weight gain, which consists of muscle, bone, water, and fat, as well as fat mass caused by the HFD. Because DEXA works by passing X-rays through the body, it does not differentiate between white adipose tissue (WAT), brown adipose tissue, and beige fat [40,41,42]. Among these fat tissues, excessive WAT accumulation with an increment in adipocyte hypertrophy results in obesity-related pathology including cardiovascular disease, type 2 diabetes, and metabolic syndrome [43]. As depicted in Figure 3, administration of ABE effectively reversed the marked increase in weight of total WAT (epididymal, retroperitoneal, and mesenteric fat) induced by HFD. Along with the decrease in WAT, hypertrophy of adipocytes, as measured by adipocyte size, was significantly reduced by ABE administration compared to that in the HFD group (Figure 3). Several studies have demonstrated that the intake of *A. bisporus* affects satiety and food intake. These findings support the conclusion that consuming *A. bisporus* can be one of the effective approaches to combating obesity [44,45]. In our study, ABE administration led to a reduction in food efficiency ratio (Table 3). The delayed fat digestion, mediated by the inhibition of lipase, is known to contribute to appetite suppression [46,47], and this aligns with ABE’s inhibitory effect on pancreatic lipase activity. In terms of hematological parameters, elevated levels of glucose, ALT, and AST in the serum induced by HFD were suppressed by ABE administration and the increased level of triglycerides caused by HFD was diminished by administration of ABE (Table 4). These findings suggest that administration of ABE can decrease body fat and weight and improve hematological parameters.

We also examined histological parameters associated with liver dysfunction in liver tissues. The liver plays a crucial role in lipid synthesis and distribution. Excessive accumulation of fats in the liver can lead to dyslipidemia and hyperglycemia, a condition known as NAFLD characterized by elevated levels of ALT and AST in serum, high fat content, and the presence of steatosis in the liver tissue [48,49,50]. H&E staining of the liver demonstrated that the high-fat diet induced hepatic steatosis, with numerous macrovesicles, lipid droplets, and hepatocellular ballooning. However, the administration of ABE ameliorated these histopathological changes (Figure 4A). Additionally, our study revealed that ABE administration significantly reduced HFD-induced lipid accumulation in the liver, as detected using oil-red O staining (Figure 4B). Weight and lipid levels of the liver are known to follow hepatic steatosis [49,51]. Our study confirmed that liver weight and levels of lipids, triglycerides, and cholesterol were dramatically increased by HFD feeding. However, such increases were reversed by the administration of ABE (Table 5). Overall, treatment with ABE and chitosan seems to exert a preventive effect on obesity-related NAFLD, as evidenced by their ability not only to alleviate ALT and AST levels in the blood but also to reduce lipid levels in the liver and improve histopathological changes associated with hepatic steatosis.

We demonstrated that administration of ABE exhibited a fat trapping ability both in vitro and in vivo. Fat trapping ability by inhibition of pancreatic lipase can be supported by an increase in fecal fat excretion, resulting from the reduced absorption of dietary lipids in the small intestine [52,53]. In this respect, the level of fecal fat excretion including lipids, triglycerides and cholesterol is a well-established method for assessing fat malabsorption [25,54]. In our study, we observed that administration of ABE upregulated the weight of total lipid and triglyceride excretion in feces compared to the HFD group (Table 6). This suggests that ABE might inhibit the level of lipid absorption not only acutely, but also chronically.

To summarize results of our study, administration of ABE rich in polysaccharides showed promising effects in preventing obesity and its associated symptoms such as hyperlipidemia, hyperglycemia, and NAFLD. These effects were supported by the ABE’s effects on the reduction in body weight gain, fat mass percentage, and food efficiency ratio with improvements in the serum glucose, ALT, AST, cholesterol, and triglyceride levels. Additionally, this treatment resulted in decreased weight of WAT and reduced lipid levels in the liver. These changes were associated with not only inhibition of pancreatic lipase activity in vitro and postprandial plasma lipids level in vivo, as observed in the OLTT, but also upregulation of fecal excretion level of lipids in the HFD model by administration of ABE. Consequently, ABE holds potential as a candidate for developing a functional food ingredient with anti-obesity properties.

## Figures and Tables

**Table 1:** The composition of control diet and high-fat diet used in this study.

Class Description	Ingredients	Control Diet (CD)	High-Fat Diet (HFD)
Protein	Casein, latic	200.00 g	200.00 g
Protein	Cystine, L	3.00 g	3.00 g
Carbohydrate	Sucrose	354.00 g	72.80 g
Carbohydrate	Starch, corn	315.00 g	-
Carbohydrate	Maltodextrin	35.00 g	125.00 g
Fiber	Cellulose	50.00 g	50.00 g
Fat	Lard	20.00 g	245.00 g
Fat	Soybean oil	25.00 g	25.00 g
Mineral	Mineral mixture ^(1)^	50.00 g	50.00 g
Vitamin	Choline bitartrate	2.00 g	2.00 g
Vitamin	Vitamin mixture ^(2)^	1.00 g	1.00 g
Dye	Dye	0.05 g	0.05 g
		Total: 1055.05 g	Total: 773.85 g

^(1)^ Mineral mixture (S10026B, Research Diets Inc.). ^(2)^ Vitamin mixture (V10001C, Research Diets Inc.).

**Table 2:** Effect of ABE and chitosan on pancreatic lipase activity in vitro.

	Inhibition of Pancreatic Lipase Activity (%)	
(mg/mL)	ABE	Chitosan
0	0 ^d^	0 ^e^
0.01	21.4 ± 2.0 ^c^	16.8 ± 2.2 ^d^
0.05	29.1 ± 2.2 ^b^	23.4 ± 1.9 ^cd^
0.1	32.2 ± 2.4 ^ab^	23.9 ± 1.7 ^bcd^
0.5	33.2 ± 2.1 ^ab^	25.4 ± 3.7 ^bc^
1.0	36.9 ± 1.7 ^a^	26.3 ± 2.7 ^abc^
1.5	35.1 ± 2.0 ^a^	29.1 ± 2.4 ^abc^
2.0	34.5 ± 1.7 ^ab^	31.5 ± 2.1 ^ab^
3.0	32.3 ± 1.7 ^ab^	33.4 ± 2.1 ^a^

Values are expressed as the mean ± SEM. Means without the same letter, *p* < 0.05.

**Table 3:** Effect of ABE administration on body weight, body composition, and food intake in HFD-fed C57BL/6N mice.

	CD	HFD	HFD + A80	HFD + A160	HFD + C160
Initial body weight (g)	19.2 ± 0.7	20.7 ± 0.4	21.1 ± 0.2	20.7 ± 0.5	21.1 ± 0.2
Final body weight (g)	29.4 ± 0.4	40.6 ± 0.7 ***^,a^	34.8 ± 0.5 ^b^	33.5 ± 0.9 ^b^	35.5 ± 1.0 ^b^
Body weight gain (g)	10.2 ± 0.9	20.0 ± 0.6 ***^,a^	13.7 ± 0.4 ^b^	12.8 ± 1.2 ^b^	14.4 ± 0.9 ^b^
Lean mass percentage (%)	75.8 ± 0.8	60.4 ±± 0.7 ***^,b^	62.4 ± 0.8 ^ab^	64.1 ± 1.0 ^a^	63.2 ± 0.8 ^a^
Fat mass percentage (%)	24.2 ± 0.9	39.6 ± 0.7 ***^,a^	37.6 ± 0.8 ^ab^	35.9 ± 1.0 ^b^	36.8 ± 0.8 ^b^
Food intake (g/day)	2.85 ± 0.04	2.46 ± 0.04 ***^,a^	2.18 ± 0.02 ^c^	2.24 ± 0.02 ^c^	2.32 ± 0.01 ^b^
Food efficiency ratio ^(1)^	0.065 ± 0.006	0.148 ± 0.005 ***^,a^	0.115 ± 0.004 ^b^	0.104 ± 0.010 ^b^	0.112 ± 0.007 ^b^

^(1)^ Food efficiency ratio = weight gain/food intake. Values are expressed as the mean ± SEM (n = 10). *** *p* < 0.001 significantly different from the CD group. Means without a common letter differ among the HFD, HFD + A80, HFD + A160, HFD + C160 group at *p* < 0.05.

**Table 4:** Effect of ABE administration on serum glucose and lipid levels and serum ALT and AST activities in HFD-fed C57BL/6N mice.

	CD	HFD	HFD + A80	HFD + A160	HFD + C160
Glucose (mg/dL)	197.2 ± 6.6	222.6 ± 8.4 *^,a^	186.3 ± 6.5 ^b^	178.0 ± 10.8 ^b^	195.6 ± 13.0 ^ab^
Triglycerides (mg/dL)	38.0 ± 2.5	54.5 ± 2.8 ***^,a^	47.2 ± 3.2 ^ab^	41.1 ± 2.7 ^b^	39.0 ± 2.3 ^b^
Total cholesterol (mg/dL)	145.4 ± 9.2	176.2 ± 3.8 **	171.2 ± 5.6	166.4 ± 9.5	160.0 ± 4.6
ALT (U/L)	51.4 ± 7.4	122.1 ± 21.6 **^,a^	70.4 ± 8.9 ^b^	55.9 ± 5.6 ^b^	90.2 ± 22.4 ^ab^
AST (U/L)	118.8 ± 12.1	192.1 ± 19.6 **^,a^	138.3 ± 9.5 ^b^	139.2 ± 11.1 ^b^	169.3 ± 17.8 ^ab^

Values are expressed as the mean ± SEM (n = 10). * *p* < 0.05, ** *p* < 0.01, *** *p* < 0.001 significantly different from the CD group. Means without a common letter differ among the HFD, HFD + A80, HFD + A160, HFD + C160 group at *p* < 0.05.

**Table 5:** Effect of ABE administration on liver weights and lipid levels in livers in HFD-fed C57BL/6N mice.

	CD	HFD	HFD + A80	HFD + A160	HFD + C160
Liver weight (g)	0.98 ± 0.02	1.14 ± 0.05 **^,a^	0.95 ± 0.05 ^b^	0.89 ± 0.03 ^b^	0.91 ± 0.03 ^b^
Lipids (mg/g liver)	84.0 ± 2.9	121.6 ± 7.4 ***^,a^	99.2 ± 3.1 ^ab^	89.6 ± 3.4 ^b^	90.3 ± 4.0 ^b^
Total lipids in liver (mg/liver)	82.5 ± 7.0	139.5 ± 14.2 **^,a^	96.2 ± 10.6 ^b^	83.6 ± 5.7 ^b^	82.1 ± 6.4 ^b^
Triglycerides (μmol/g liver)	19.8 ± 0.7	24.6 ± 1.0 **	23.6 ± 1.1	22.9 ± 0.8	23.8 ± 1.3
Total triglycerides in liver (μmol/liver)	19.7 ± 1.2	26.6 ± 2.1 *^,a^	23.5 ± 2.0 ^ab^	20.8 ± 1.2 ^b^	21.5 ± 1.3 ^b^
Cholesterol (μmol/g liver)	5.4 ± 0.3	7.4 ± 0.5 **	7.3 ± 0.4	6.7 ± 0.4	6.8 ± 0.4
Total cholesterol in liver (μmol/liver)	5.3 ± 0.3	8.5 ± 1.1 **^,a^	7.0 ± 0.7 ^ab^	6.0 ± 0.4 ^b^	6.2 ± 0.4 ^b^

Values are expressed as the mean ± SEM (n = 10). * *p* < 0.05, ** *p* < 0.01, *** *p* < 0.001 significantly different from the CD group. Means without a common letter differ among the HFD, HFD + A80, HFD + A160, HFD + C160 groups at *p* < 0.05.

**Table 6:** Effect of ABE administration on fat excretion in feces of HFD-fed C57BL/6N mice.

	CD	HFD	HFD + A80	HFD + A160	HFD + C160
Lipids (mg/g feces)	23.41 ± 1.68	35.40 ± 2.53 ***	37.48 ± 1.84	40.76 ± 2.86	39.70 ± 2.14
Total lipids in feces (mg/day)	6.49 ± 1.05	12.64 ± 0.89 ***^,b^	12.47 ± 0.86 ^b^	18.32 ± 1.93 ^a^	15.50 ± 1.88 ^ab^
Triglycerides (μmol/g feces)	14.86 ± 0.93	17.08 ± 1.12	18.68 ± 1.18	19.22 ± 1.73	17.95 ± 1.07
Total triglycerides in feces (μmol/day)	4.89 ± 0.61	5.30 ± 0.38 ^b^	5.77 ± 0.42 ^b^	7.70 ± 0.67 ^a^	6.90 ± 0.83 ^ab^
Cholesterol (μmol/g feces)	4.81 ± 0.42	5.40 ± 0.40	5.76 ± 0.31	6.05 ± 0.47	5.92 ± 0.29
Total cholesterol in feces (μmol/day)	1.46 ± 0.24	1.88 ± 0.15	1.88 ± 0.17	2.40 ± 0.16	2.42 ± 0.28

Values are expressed as the mean ± SEM (n = 10). *** *p* < 0.001 significantly different from the CD group. Means without a common letter differ among the HFD, HFD + A80, HFD + A160, HFD + C160 groups at *p* < 0.05.

## Data Availability

The data will be made available upon request.
